# Microplastics: A Real Global Threat for Environment and Food Safety: A State of the Art Review

**DOI:** 10.3390/nu15030617

**Published:** 2023-01-25

**Authors:** Khaled Ziani, Corina-Bianca Ioniță-Mîndrican, Magdalena Mititelu, Sorinel Marius Neacșu, Carolina Negrei, Elena Moroșan, Doina Drăgănescu, Olivia-Teodora Preda

**Affiliations:** 1 Department of Clinical Laboratory and Food Safety, Faculty of Pharmacy, “Carol Davila” University of Medicine and Pharmacy, 020956 Bucharest, Romania; 2 Department of Toxicology, Faculty of Pharmacy, “Carol Davila” University of Medicine and Pharmacy, 020945 Bucharest, Romania; 3 Professional Farma Line, 105200 Baicoi, Romania; 4 Department of Pharmaceutical Physics and Informatics, Faculty of Pharmacy, “Carol Davila” University of Medicine and Pharmacy, 020956 Bucharest, Romania

**Keywords:** plastic pollution, plastic waste, sources of microplastics, ecotoxicity, food safety, public health, biodegradable materials

## Abstract

Microplastics are small plastic particles that come from the degradation of plastics, ubiquitous in nature and therefore affect both wildlife and humans. They have been detected in many marine species, but also in drinking water and in numerous foods, such as salt, honey and marine organisms. Exposure to microplastics can also occur through inhaled air. Data from animal studies have shown that once absorbed, plastic micro- and nanoparticles can distribute to the liver, spleen, heart, lungs, thymus, reproductive organs, kidneys and even the brain (crosses the blood–brain barrier). In addition, microplastics are transport operators of persistent organic pollutants or heavy metals from invertebrate organisms to other higher trophic levels. After ingestion, the additives and monomers in their composition can interfere with important biological processes in the human body and can cause disruption of the endocrine, immune system; can have a negative impact on mobility, reproduction and development; and can cause carcinogenesis. The pandemic caused by COVID-19 has affected not only human health and national economies but also the environment, due to the large volume of waste in the form of discarded personal protective equipment. The remarkable increase in global use of face masks, which mainly contain polypropylene, and poor waste management have led to worsening microplastic pollution, and the long-term consequences can be extremely devastating if urgent action is not taken.

## 1. Introduction

Synthetic polymers appeared in the late 19th century around the 1860s, but it wasn’t until after World War II that the “plastics boom” really began. Plastic has become one of the most widespread materials since its beginnings as a phenol-formaldehyde resin (i.e., Bakelite) [1]. At its core, plastic was designed to improve human living conditions, but today it has become a real danger to the environment and the safety of the planet [2].

Nowadays, plastic is ubiquitous in all compartments of the environment (air, water and soil), especially due to the fact that the food packaging found on the market for food products such as dairy products, meat, fish or drinks, including mineral water, are made in large part from plastic. Contact between food and plastic packaging is almost always the cause of mutual transfers between container and contents. The quality of food products is influenced by the contamination resulting from the interaction with the substances in the composition of the packaging, sometimes the alteration of the nutritional qualities being doubled and affecting the safety of consumption [3]. The presence of microplastics has been detected in soil ecosystems, surface waters [4], coastal sediments [5], beach sands [6], freshwater sediments [7] and deep environments [8]; even rain and snow contain significant numbers of microplastics that can sometimes be invisible to the naked eye. Indeed, the intensive exploitation of plastic associated with the poor performance of waste management systems, including end-of-life collection and capture, has led to a massive accumulation of plastic waste in the environment. The release of plastic materials into the environment is recognized as an important pollution problem [9,10].

The increasing presence of microplastics in the environment is causing serious pollution worldwide. Due to their characteristics, namely, synthetic materials with high polymer content, solid particles smaller than 5 mm, insoluble in water and non-degradable, microplastics are easily introduced into the environment and persist there for a long time [11]. Food chains suffer major pollution due to emissions of hydrophobic organic chemicals [12,13,14,15,16]. Being present in various aquatic ecosystems (surface waters, oceans, estuarine waters, etc.), marine organisms are directly or indirectly exposed to microplastics. The scientific literature reports the negative impact of microplastics on benthic organisms [17,18]. The toxic effects of these pollutants have been studied on the feeding patterns, growth and reproductive systems of several aquatic species [19,20,21,22,23]. Therefore, humans are exposed to these pollutants through the consumption of seafood, fish and crustaceans.

The degradation of plastic waste generates microplastic (MP) or nanoplastic (NP) particles. This division is based on the diameter of the plastic fragments or particles, with MPs having a diameter of less than 5 mm and NPs having a diameter of 1 to 100 or 1000 nm [24]. About the diameter of plastic particles, the scientific literature provides more information and divisions of microplastics. When first reported in 2004, the term “microplastic” was used to describe fragments of plastic approximately 20 μm in diameter. However, while these early reports referred to truly microscopic particles, they did not provide a specific definition of microplastics. In 2008, the U.S. National Oceanographic and Atmospheric Agency (NOAA) hosted the first international workshop on microplastics in Washington, U.S.A., and as part of this meeting, formulated a broader working definition to include all particles smaller than 5 mm. Other authors consider particles >5 mm as macroplastics, mesoplastics from 5 to >1 mm, microplastics from 1 mm to >0.1 μm, and nanoplastics as 0.1 μm [25]. Microplastic samples are usually sorted into different forms depending on the observed morphology [26]. According to Lambert et al., “microplastics” is an umbrella term that covers many particle shapes, sizes and polymer types, and as such, the physical and chemical properties of environmental microplastics will differ from the primary microbeads commonly used for ecotoxicity testing [27].

The purpose of this article is to highlight the magnitude of the danger of microplastic contamination of the environment with its negative effects on the food chain and implicitly on human health, thus drawing attention to the need to intensify efforts to stop the increase in pollution of these particularly versatile and toxic contaminants. A detailed presentation of these contaminants, correlated with the categories of food products most exposed to the danger of contamination and with the disruptive mechanisms at the level of live food sources but also in the body, helps to create a more complete picture of the devastating effects of microplastics for the environment and the safety of food intended for human consumption.

There is sufficient evidence of the negative effects caused by microplastics on living organisms, especially on aquatic species that are otherwise considered valuable sources of nutrients for the human body. As a result, the accumulation of microplastics in marine organisms represents, on the one hand, a danger for these nutritious species and, on the other hand, a danger for the health of the consumer who feeds on contaminated species. Unfortunately, contamination with microplastics has become evident in many categories of food products, including some that are consumed frequently and in large quantities, namely, drinking water and kitchen salt.

## 2. Materials and Methods

In order to create an overview related to the danger of microplastic pollution on the environment and human health, databases were accessed using the following keywords: ecotoxicity, microplastics in health, microplastic pollution, sources of microplastics, plastic waste, the toxicity of microplastics, microplastics in the marine environment, microplastics in food. More than 6900 articles were identified, of which over 250 were included in the present study, as follows: ScienceDirect 169 total results (Marine Pollution Bulletin—61, Journal of Hazardous Materials—52, Current Opinion in Environmental Science & Health—39, Ecotoxicology and Environmental Safety—17), ACS PUBLICATIONS (American Chemical Society) 119 total results (Contaminants in Aquatic and Terrestrial Environments—40, Ecotoxicology and Human Environmental Health—40, Perspectives—15, Ecotoxicology and Public Health—13, Critical Reviews—11), WEB OF SCIENCE Core Collection (without refining) 1905 total results (193 results from Web of Science Core Collection—Highly Cited Papers), NCBI (National Library of Medicine) (2007–2022) 1775 total results and SPRINGER 2936 total results (Materials Science—1274, Environment, general—874, Ecotoxicology—788).

## 3. Sources of Microplastics

Inadequate management of plastic waste has led to increased contamination of freshwater and marine environments.

Plastic materials represent between 60% and 80% of the waste present in the marine environment and 90% of the waste floating on the seas and oceans [28]. Plastic waste present in the marine environment is a threat to both the environment and marine fauna due to the risk of being swallowed by marine life. Statistics show that at least 267 species worldwide are affected by this problem, including 44% of birds, 43% of mammals, 86% of turtles and various fish species [29]. Plastic waste has a negative impact on the health of marine ecosystems as evidenced by the increasing number of marine species affected. These fragments of plastic material, decomposed into microparticles in suspension in the water column, or deposited in sediments, slow down or prevent the vertical transfer of oxygen [30].

In a recent study [31], it is specified that people consume an average of 39,000–52,000 microplastic particles per year. This result was obtained on the basis of studies in which the amounts of microplastics that different foods contain are evaluated. If the estimates for inhaled microplastic particles are added, the number can rise to around 74,000 particles. For drinking tap water, another 4000 particles are added, while for drinking water bottled in plastic, the number increases by 9000 particles. The author of the study Kieran Cox believes that these data underestimate the actual consumption of microplastics, and it is possible that in reality the values are much higher.

The main route of human exposure to MPs is through food ingestion, including seafood contaminated with microplastics [32], commercial processed fish [33], sea salt [34], honey, beer and food components. Most of these food products are also sometimes contaminated by the presence of impurities from processing materials and contaminants present in the packaging [35]. The second route of exposure is through the inhalation of air and dust containing MPs [32]. Given their high nutritional value, seafood plays an important role in the human diet, and therefore the consumption of these contaminated foods represents an increased health risk, especially for small fish eaten whole [36]. Microplastics come from a range of polymeric materials mixed with various additives manufactured as such or result from the degradation and fragmentation of plastic waste into microparticles. Their behavior in the oceans is similar to that of marine plankton, and they can be ingested by aquatic fauna, being mistaken for small natural prey and consumed by filter-feeding species, such as bivalve mollusks, or adsorbed on macroalgae. These microplastics are potential carriers of chemical contaminants, such as the additives that constitute them and the persistent organic pollutants that are adsorbed and concentrated in them. An entire microbial community, called the “plastisphere”, also develops there, including bacteria of the genus *Vibrio* spp. [37].

Several studies have highlighted the presence of microplastics in numerous commercial aquatic species such as mussels, oysters, crabs, shrimps and fish [38,39]. The results of these studies suggest that humans are exposed to microplastics through the consumption of contaminated aquatic species, and the presence of microplastics in seafood could pose a threat to food safety. The potential accumulation of microplastics in food chains, particularly in fish and crustaceans, appears to be the main source of human exposure to microplastics. Contamination of foodstuffs with MPs could have consequences for the health of human consumers. In this trophic context, the fate and toxicity of microplastics in humans constitutes a major knowledge gap that deserves special attention.

The passage of microplastics from the intestine into the circulatory system and various tissues and cells in humans has been studied by several authors [40], and it has been observed that the absorption of MP particles from the gastrointestinal tract into the lymph and circulatory system occurs.

The toxicological effects of ingesting nano- and microplastics present in sea food products are still controversial and cannot be assessed at the current level of knowledge, which is still limited for these emerging dangers [41,42].

As previously stated, microplastics are generally defined by their size, shape and color [43,44]. Definitions differ between research teams, but five categories are generally used to describe the form of microplastics: fragments, spheres, fibers, granules (or pellets) of industrial plastic and “foam” (which refers to fragments of expanded polystyrene). The origin of microplastics in the aquatic environment is divided into two major sources: the first source of microplastics (primary microplastics) corresponds to plastics produced directly as microparticles, and the second source (secondary microplastics) corresponds to the fragmentation of larger plastic debris (>5 mm) through a combination of physical, chemical and biological processes [45]. This distinction enables the potential identification of certain sources of microplastics, making it possible to act upstream to reduce their presence in the environment.

Depending on their origin, microplastics can be classified into two categories: primary or secondary microplastics [46]. Primary microplastics are microscopic synthetic polymers used as exfoliants in various processes: chemical formulations, grinding products, maintenance of various plastic products and manufacturing of synthetic clothing. Microbeads are another type of primary plastic (size < 2 mm). These are polyethylene (PE), polypropylene (PP) and polystyrene (PS) beads used in cosmetics and care products [47]. All these microplastics can have implications in the biomagnification and bioaccumulation of various chemicals and pollutants due to their high surface area/volume [48].

There are studies that have shown that a facial exfoliator can discharge between 4594 and 94,500 PE microspheres (average diameter: 164 and 327 µm) into wastewater during a single use. Due to their very small size (often < 100 µm), these microparticles can pass whole or in part through the various treatments of wastewater treatment plants before reaching the aquatic environment [49,50,51,52]. Washing machines also represent, through discharges from sewage treatment plants, a large primary microplastic contribution to the aquatic ecosystem. Synthetic fibers appear to be ten times more abundant than cosmetic microplastics and washing a single piece of synthetic clothing can release over 1900 strands (polyester, acrylic, polyamide). Another important source of primary microplastics is the supply of synthetic fibers into the aquatic environment [53,54]. Microplastics have also taken the place of sand in blast cleaning [55].

Secondary microplastics are the fragmented product of macro- or mesoplastics, mainly under the effect of various environmental processes: biodegradation, photo-degradation, thermo-oxidative degradation, thermal degradation and hydrolysis [56].

Microplastics found in so-called secondary aquatic environments originate from the fragmentation/degradation of macroplastics already present as waste in the environment. Degradation of polymers corresponds to the alteration of their properties or molecular structure that can cause fragmentation, unlike mineralization which is a complete degradation of polymers following the destruction of carbon chains that leads to their conversion into small molecules of carbon dioxide or methane. Very few plastics can be mineralized in the aquatic environment, the main representatives being certain biopolymers and aliphatic polyesters. Fragmentation of polymers occurs following a weakening of their structural integrity and generates smaller particles, including microplastics [57,58]. Fragmentation depends on the nature of the particles and their degree of crystallinity. The latter is at the origin of the number and surface area of pores in polymers and thus influences their stability, affecting their physical properties and water permeability. Photo-degradation is the main cause of degradation of polymers present on beaches or on the water surface and is due to exposure to UV radiation [59,60]. This process generates oxidation reactions that will initiate the destruction of the C-H chemical bonds of the polymer chain and the formation of free radicals. These will react with oxygen and form peroxide radicals.

Other polymers (PE, PP, PC—polycarbonate) also turn yellow upon exposure to ultraviolet (UV) radiation, but the mechanisms remain poorly understood. This yellowing phenomenon can also be due to the degradation of additives present in the polymer matrix, such as phenolic antioxidants whose degradation products contain quinoid structures that can cause this coloring [61].

Apart from photo-degradation, other processes are, to a lesser extent, responsible for the degradation of polymers, including biodegradation (action of microorganisms), hydrolysis and finally erosion by mechanical processes [62,63,64].

The chemical elements that make up the polymers can become a source of environmental contamination [65,66]. Polymer matrices are a mixture of monomers associated with initiators and catalysts necessary for the polymerization process. Additives are then added to the mixture to give the polymers the desired properties. All these chemical compounds can be found free in the polymer matrix during polymerization reactions that are rarely completely completed or during polymer degradation. Free, they can migrate into the environment throughout the life cycle of polymers and end up in aquatic systems [67,68,69,70].

Most of these molecules are environmentally toxic; some are known reproductive toxicants (BPA—bisphenol A, styrene, phthalates) and/or probable or known carcinogens (BPA, styrene, vinyl chloride) [71,72,73,74,75]. In addition, they tend to persist in the aquatic environment due to their low biodegradability and be bioaccumulated by marine organisms. These chemical compounds were detected in aquatic biomes: pelagic zone [76,77], intertidal zone [78], benthic zone [79,80] and within biological communities [81,82,83,84,85].

### 3.1. Primary Microplastics

Primary microplastics with a diameter of less than 5 mm are intentionally manufactured by the plastics industries [86]. This small size makes it possible, among other things, to control the viscosity, stability and physical appearance of the product or even to have an abrasive effect. Primary microplastics are found in many cosmetics, cleaning products, artificial turf and fishing nets.

Primary microplastics may also be found in industrial products, albeit unintentionally. Despite this, the result remains the same; microplastics end up in the environment. The four main sources of these are, in descending order of importance, car tires, paint-based markings, plastic resins and synthetic clothing in the form of synthetic fibers. The rubbing of tires against roads during acceleration and braking or when roads are very abrasive can lead to tire wear and thus the microplastics used in tire composition come free and end up on the road. These can then be carried along with rainwater into purification systems. Microplastics are not retained by the purification system because it is not designed to retain such small particles. Consequently, they are released into the waters. The same is true for microplastics in the form of synthetic fibers found in synthetic clothing. In the study by Brown et al. (2011), the authors found that synthetic clothing is a major source of microplastics in the marine environment. These fibers are mainly made up of polyester (78%) and acrylic fibers (22%). Most of the synthetic fibers that have been found in marine sediments and sewage effluent closely resemble the fibers used in synthetic clothing. They therefore analyzed the effluents of washing machines with and without clothes and found that more than 100 fibers were released per liter of effluent. A single synthetic garment can release up to 1900 synthetic fibers in a single machine [87].

### 3.2. Secondary Microplastics

In the environment, especially in the ocean, plastic faces mechanical, physical and biological forces. Indeed, following oxidation by UV rays, low temperatures as well as mechanical abrasion from waves and sand, plastic degrades and is reduced to scrap. Secondary microplastics are thus derived from the fragmentation of macroplastics. Despite warnings about the danger of microplastic pollution, in 2019, of the approximately 370 million tons of plastic produced worldwide, only 9% were recycled, 12% incinerated and the rest ended up in the environment or landfills [88]. All these wastes represent a major source for the formation of secondary microplastics.

**Figure 1:**
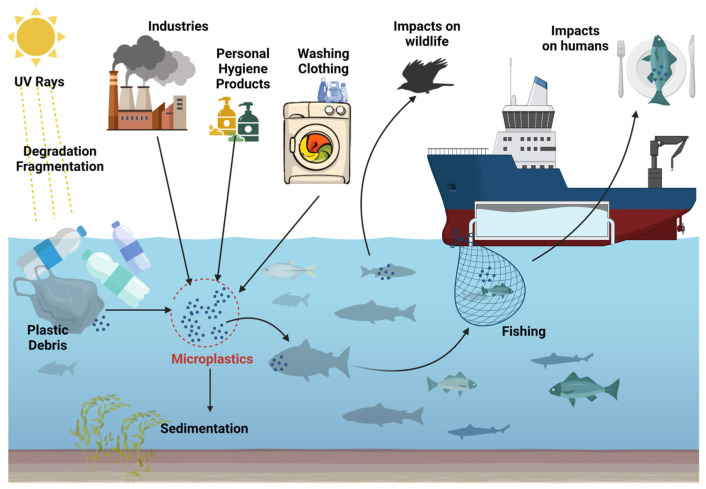
Microplastics in aquatic environment. Created with BioRender.com.

Natural catastrophes like hurricanes or floods can hasten the transfer of garbage from the land to the maritime environment. Ships, commercial and recreational fishing, tourism, aquaculture and maritime businesses, such as oil rigs, can all be direct causes of microplastic pollution in the ocean, endangering both marine life and vegetation. (Figure 1). As a result of tourism and leisure activities, a wide range of plastics remains, which is dumped on the beach or in coastal resorts. Discarded or lost fishing gear, such as monofilament lines or nylon nets, are the most common plastics, constituting a source of marine pollution.

### 3.3. Distribution of Microplastics in the Environment

Microplastic particles can be found everywhere in the environment. Indeed, they can be found in the air, drinking water, food we eat and even in lakes, rivers and seas; they are all around us. Wastewater treatment plants are not suitable for filtering such small particles and thus contribute to their release into the environment. Their transport will depend on the size, shape and density of the polymer. The density of the polymer will determine whether or not it floats on the surface of the seawater. Density is the relationship between the mass of the body and its volume: ρ = m/V. It is expressed in g/cm^3^ according to the International System (I.S.) [89].

Since the density of seawater is about 1.02 g/cm^3^, polymers with a lower density will float on the surface and those with a higher density will sink. The latter will settle to the bottom of oceans, lakes and rivers and join the benthic fauna and flora. Among the most common polymers, only polyethylene (PE), polypropylene (PP) and polystyrene (PS) will float. Instead, polyvinyl chloride (PVC), polyamide (PA) and polyethylene terephthalate (PET) will sink [90].

**Figure 2:**
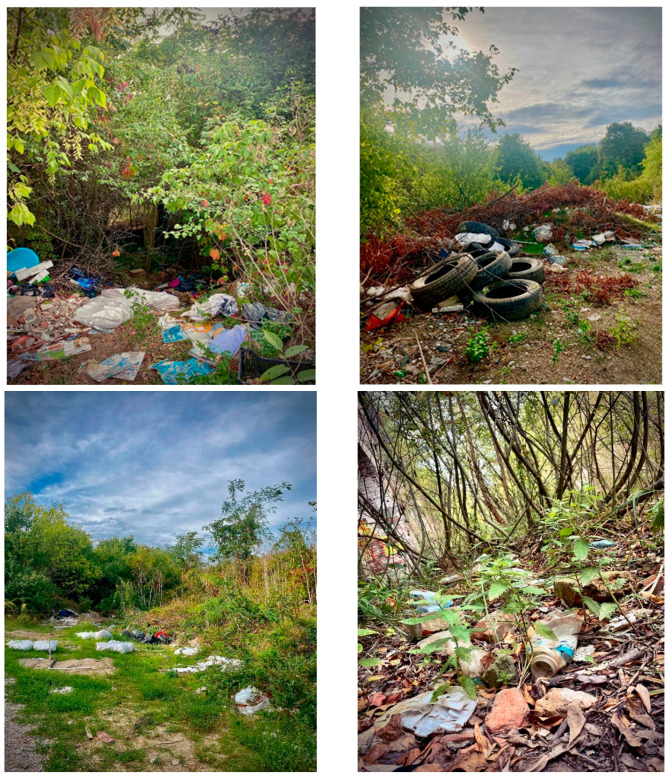

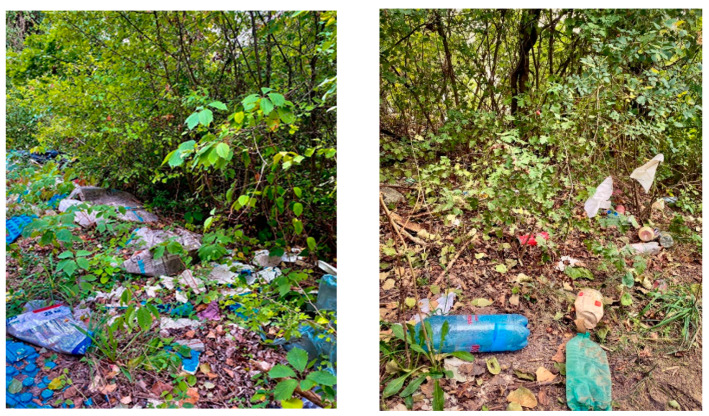
Sources of microplastic contamination (original photographs taken by the authors in isolated locations from Fortul I, Chitila 44°29′46″ N 25°59′12″ E in Romania on 17 September 2022).

Microplastics have also been found in the Arctic Ocean, as it appears from a study published in 2015 [91]. Samples of water from the Arctic Ocean were collected and analyzed from the surface (16 cm below the water surface) but also from deeper areas (6 m below the water surface). On the surface, 20/21 samples were contaminated with an average of 1.31 particles/m^3^ or 1310 particles/liter. At depth, 70/75 samples contained microplastics, with concentrations between 0 and 11.5 particles/m^3^. Among the types of plastic found, most were represented by fibers (95%). Fourier transform infrared spectrometry (IRTF) made it possible to differentiate the polymers that make up these fibers. Thus, polyester, nylon, acrylic fibers and polyvinyl chloride, as well as microplastics of unknown origin, were identified. These polymers are of high density and over time will sink and sediment on the seabed. Their presence on the surface of the water could also come from the turbulence caused by winds and storms that lead to the redistribution of particles in the water column. Contamination with local microplastics cannot be excluded [91]. In short, microplastics are found even in places isolated from human activities (Figure 2).

## 4. Problematic Management of Plastic Waste

Despite the announced success of plastics 70 years ago, the means to manage the production, use and therefore end-of-life of the waste were not anticipated [92].

In the current economic model, the low cost of plastic production does not reflect the high cost of recycling or waste disposal as it is not borne by the producer or the consumer. Furthermore, the lifestyle of modern societies is not consistent with a policy of limiting the production of plastic waste. For example, over 50% of the plastics produced are single-use or short-lived (e.g., packaging, food bags) and only 20% are long-lived (e.g., pipes and other construction components) [93,94]. The remaining 30% are intermediate-life plastics used in electronics, automotive or furniture design. To date, there are three options for plastic at the end of its life cycle: (1) incineration (the only method of complete destruction) with or without recovery of the energy produced in electricity and heat; (2) recycling and recovery of raw material; (3) and disposal in landfills or in the environment. In total, since 1950, 79% of the plastics produced have been deposited in landfills or in the environment [95].

The disposal (accidental or voluntary) of plastic waste in the environment is extremely problematic as none of the main polymers used by the plastics industry are biodegradable; so, they tend to accumulate. This accumulation is already sufficient to allow plastics to be used as a marker of the Anthropocene [95,96]. In 2015, 47% (60–99 metric tons) of plastic waste worldwide was deposited in the environment. However, depending on the region of the world, the fraction of improperly managed waste is particularly uneven [97]. Europe and North America release between 1% and 10% of the waste they produce, while these percentages are much higher in Asia (63%) and Africa (85%). However, it is important to note that some of the waste generated in Europe and North America is exported to Asia and Africa for end-of-life treatment. For example, China has received 45% of all waste generated since 1992 [98]. With the increase in human population and therefore demand, the amount of plastic waste produced could reach 380 metric tons by 2060 [99]. According to the three scenarios defined by these authors, the amount released annually from 2060 would reach an average value of 213 metric tons in the most extreme case, with Africa and Asia being the main contributors.

### 4.1. The Influence of Plastic Waste on Human Society

Unlike many chemical pollutants, plastic waste receives special attention from the media for simple reasons: it comes from people’s daily uses, it is visible, it accumulates, and it has an impact on several economic sectors [100]. Two areas are mainly affected by plastic waste emissions: tourism and fishing/aquaculture. The main economic loss is related to coastal and/or infrastructure cleanup. The presence of coastal debris reduces the attractiveness of a region to tourists, influencing local economies (e.g., hotel prices and attractions) [101]. Thus, to avoid economic decline, municipalities have to clean beaches and treat waste, which is not negligible and represents EUR 19 million per year for the United Kingdom [102]. For fisheries/aquaculture, the economic effects are mainly associated with the repair of equipment damaged by the presence of litter and cleaning at harvesting sites (approximately EUR 156,000 per year in Scotland) or the loss of biological resources due to debris [102]. The presence of abandoned fishing traps in the Salish Sea (U.S.A.) resulted in a 4.7% (EUR 586,000) decrease in annual crab catches [103]

Despite the lack of toxicological data, the misuse of plastics draws the attention of scientists to human health problems. Plastic particles are found in a large number of food products, such as bottled water [104], tap water [105], table salt [105,106,107], beer and canned food [108]. Based on the analysis of 159 samples of tap water, 12 brands of beer and 12 brands of table salt, Kosuth et al. [107] estimate that the annual intake of plastic particles through these products is 5800 plastic particles per capita. In addition, through seafood contamination [36], the number of plastic particles ingested annually by a consumer who habitually consumes large amounts of seafood has been estimated at 11,000.

There is also a risk of ingestion by inhalation, especially synthetic microfibers, which due to their small size are very volatile and can easily enter the respiratory tract [109,110,111]. If the different possible routes of ingestion (e.g., food, inhalation) are compiled, a human being would consume between 74,000 and 121,000 plastic particles each year [101]. In addition to particle ingestion, the French National Agency for Food Safety, Environment and Health at Work (ANSES) is interested in the toxic effects associated with additives incorporated into plastics. Indeed, additives represent, on average, 7% of the mass of a plastic [112]. However, many plastics are in direct contact with food (e.g., meat, cheese, fruit and vegetables, fish) and additives could migrate from the packaging to the product. The analysis of 120 food packages showed the presence of more than 100 chemical compounds, of which 4 were identified as potentially dangerous: DEHA (Bis(2-ethylhexyl) adipate), DEHP (Bis(2-ethylhexyl) phthalate), 2,4-DTBP (2,4-di-tert-butylphenol) and ethylene bis stearamide [98].

### 4.2. Detection of Microplastics

Different methods are used to detect microplastics. First, the samples are examined visually for particles with a size of 1–5 mm or under a microscope after taking water and sediment samples. This remains the simplest and most affordable method. However, there are new, more precise techniques: Raman spectroscopy or Fourier transform infrared (FTIR) spectroscopy.

**Figure 3:**
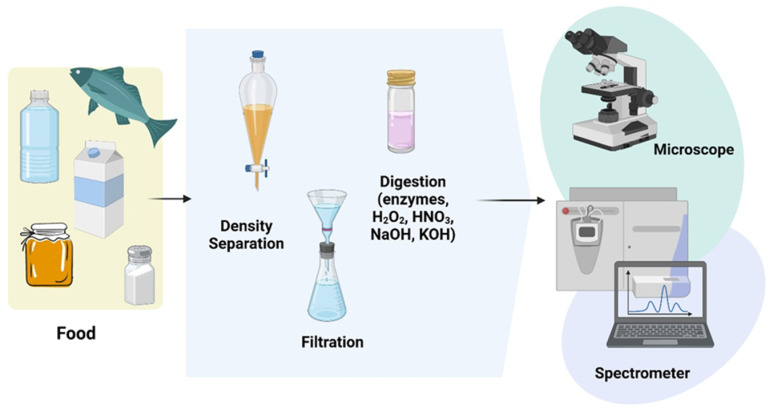
Microplastics detection stages. Created with BioRender.com.

The detection of microplastics involves certain difficulties (Figure 3): capturing the plastic particles, separating the plastic fragments from the other particles in the sample and, finally, identifying the type of plastic [112,113].

In the sediment analysis process, plastic particles are first sorted by size. This is done by sifting and filtering. The pores of filters generally measure between 1 and 1.6 µm and those of sieves vary between 0.038 and 4.75 mm. Density differences are then used to separate particles originating from sediments from those originating from water. Those of low density float on the surface of fresh water and sea water. These are polystyrene, polyethylene and polypropylene. In contrast, the high-density ones come from the sediments that line the sea floor and include polyvinyl chloride (PVC), nylon or polyamide (PA) and polyethylene terephthalate (PET). After separating the particles according to their size, they are sorted with the naked eye or under a microscope. This step is essential to separate them from other materials such as organic waste but also to classify them according to their nature. The separation of particles is done according to their shape, color, degradation stage and source [91].

Apart from this, Raman spectroscopy or Fourier transform infrared spectroscopy (FTIR) are the techniques most commonly used in studies for the precise identification of microplastics. They detect a size limit of about 10 µm. Fourier transform infrared spectroscopy sends infrared rays at microplastics and analyzes the radiation returned. Particles will absorb and reflect different wavelengths depending on their composition [114]. Then, the infrared spectrum of the sample is compared with the spectrum of known polymers. FTIR also makes it possible to determine the chemical composition of the particles. It is an accurate and reliable method of polymer identification. Raman spectroscopy is a technique that exposes samples to laser light. The laser light irradiates the molecules and is then scattered in different directions. It thus provides information about the structure of the polymer. It is a non-invasive technique that can be applied directly to the filter containing the particles [115]. This avoids different sampling steps that can be a source of cross-contamination. There are other detection methods, but the ones detailed above are the most commonly used. Among all these microplastic detection techniques, an important thing to consider is the minimization of cross-contamination. As microplastics are present in the ambient air or in the materials used, they can thus contaminate the samples during their analysis. This can lead to the generation of false positive results.

Therefore, it is important to ensure a clean environment for handling samples, as well as avoiding the use of plastic during the analysis process. For example, some scientists wear laboratory aprons made of natural materials and cotton. It is also recommended to disinfect the laboratory surfaces with 30% ethanol as well so as to make procedural blanks for each stage of sample processing. In the study undertaken by Mintening in 2019, blank procedures were performed using 150 L of drinking water pre-filtered through 3 µm filters. The particles were detected using Fourier transform infrared spectroscopy in two of four samples. For the rest of the study, the scientists considered the average contamination of the procedural blanks when analyzing the samples. Therefore, it is important to perform these procedural blanks and to take into account cross-contamination in order not to falsify the results of the analysis [99].

## 5. Potential Effects of Microplastics on Human and Animal Health

Numerous studies demonstrate the presence of microplastics in food and mineral water (Table 1).

However, the question arises whether this contamination occurs before or during the food packaging or preparation process. The same is true for water. There are many dilemmas regarding microplastics that come from plastic bottles [137].

### 5.1. Mineral Water

Very recently, microplastics have been detected in mineral water contained in plastic bottles. A quantitative study estimated the concentration and number of microplastics smaller than 10 µm contained in 500 mL plastic bottles. These bottles are made of polyethylene terephthalate (PET). The scientists analyzed plastic water bottles from 10 different brands. For each brand, 3 bottles were selected, i.e., a total of 30 bottles analyzed. In parallel, three procedural blanks were carried out. The results show the presence of microplastics in all samples. The concentration varies between 3.16 +/− 0.7 particles/L and 1.1 +/− 0.8 particles/L. It is believed that hard plastic bottles would release larger sized fragments [138] as opposed to easily deformable bottles with an alkaline pH which would release microplastics of smaller sizes but in greater numbers. The most contaminated mineral water is that from plastic bottles of poor quality, i.e., thin and easily deformable [87].

Because of their presence in a large part of the water column, microalgae can interact with a large number of particulate materials of different densities [139]. In addition, microalgae can adsorb certain small microplastics on their surface and these can cover their pores, leading to a limitation of energy, O_2_/CO_2_ and nutrient transfers [140].

### 5.2. Food Salt

New research has shown that microplastics are present in the vast majority of brands of table salt sampled in Africa [102]. According to a new study by researchers in South Korea, 36 out of 39 brands of salt tested had microplastics in them. Using previous studies on salt, this new effort is the first of its kind to examine the geographic distribution of microplastics in table salt and its correlation with where plastic pollution is found in the environment [141].

The Mediterranean ecosystem is one of the most heavily polluted with plastic, heavy metals, etc. According to a study of 16 brands of table salt marketed in China, the content of MP particles was 550–681 elements/kg in sea salt, 43–364 elements/kg in lake salt and 204 elements/kg in rock salt [135]. The most common plastic is polyethylene (22.9%) and polypropylene (19.2%) According to another studies, salts are not only contaminated by aquatic sources; in addition, there is a high risk of MP contamination of table salt during the manufacturing process [142,143].

### 5.3. Bee Honey

**Figure 4:**
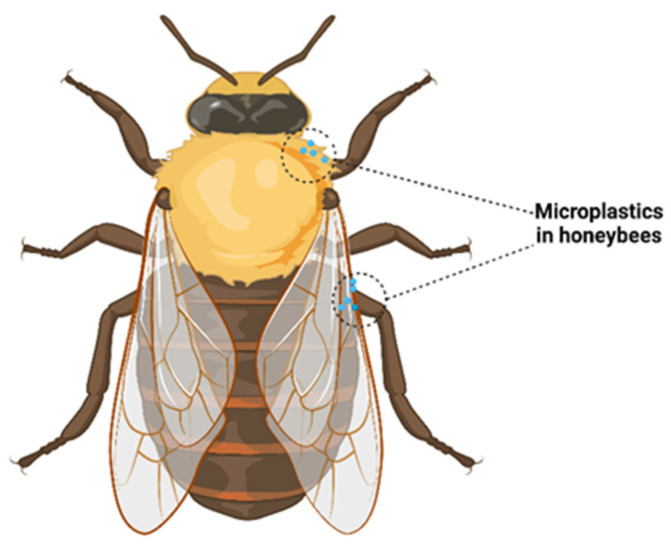
The presence of microplastics on the surface of a bee’s body, especially on the edge of the wings and the head. Created with BioRender.com.

The environment is increasingly invaded by microplastics; they have become ubiquitous, and their presence can be signalized in ecosystems that few think about. An example is to be found in bees for which it has been observed that they can be samplers of microplastics through their presence on the surface of the animal’s body, especially on the edge of the wings and head (Figure 4). For a very long time, bee products and bees have been analyzed in laboratories with the aim of using them as bioindicators for polluting agents. Due to their increased sensitivity and large flight area, bees are considered to be potential models for monitoring environmental quality. In this way, the degree of pollution present in a certain region is evaluated but also the type of pollutant that predominates (pesticides, insecticides, heavy metals). Bees, through their ability to fly many kilometers, penetrate hard-to-reach places and come into contact with all elements of the environment (from the nectar of flowers to the air) to bring pollutants into their hive, where eventually they accumulate and end up in honey and other beehive products. Various polymers have been identified, the most common of which was polyester [144].

**Figure 5:**
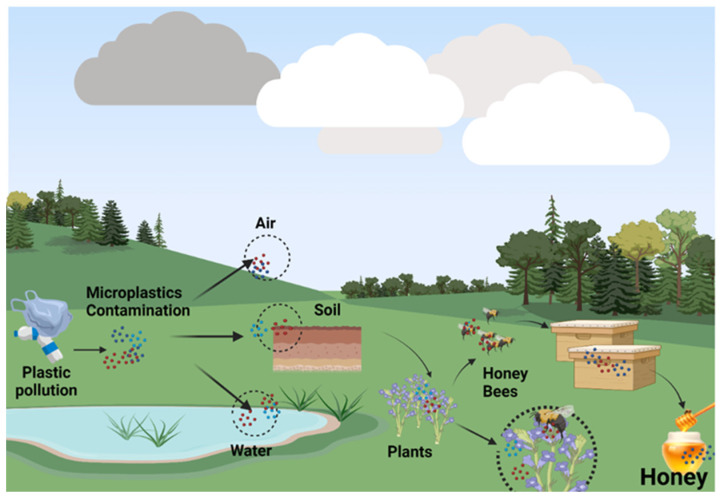
Microplastics in the terrestrial environment and the influence on apicultural products. Created with BioRender.com.

Even if for a long time it was considered that plastic, implicitly microplastics, has dimensions that cannot cross the physical barriers of intact plant tissue, studies have shown that microplastics and nanoplastics have the significant potential to contaminate plants, for example, wheat (*Triticum aestivum*) and lettuce (*Lactuca sativa*). The small particles penetrate through the roots to the shoots, reaching nectar and pollen [145]. It is believed that the presence of microplastics can prevent the proper absorption of nutrients and water by plant roots. Thus, microplastics can change the biomass of plants, the characteristics of the roots, but also the microbial activities at the soil level. The smaller the plastic particles, the greater the animal exposure; for example, in the soil, ingesting larger amounts of microplastics could cause damage to the intestinal tract of earthworms and thus reduce their survival. This process could introduce microplastics into the food chain (Figure 5) implicitly affecting not only humans, bees and plants but also the entire soil ecosystem [146].

In the analysis of 19 honey samples from different geographical areas (France, Italy, Germany, Spain, Mexico), non-pollen particles were identified in all the samples studied. Both colored and transparent fibers and particles were detected. To differentiate natural fibers such as cellulose or chitin fibers from synthetic fibers, they were subjected to fuscine and rose bengal staining. Fibers and fragments that are not colored by fuchsia and rose bengal are considered synthetic polymers. The non-pollen particles in all the samples taken in the study had an average fiber value of 166 ± 147/kg honey, and other types of fragments had a number with an average value of 9 ± 9/kg honey [147].

Likewise, another study conducted on 47 honey samples from supermarkets but also purchased directly from beekeepers in Germany, reported the presence of fibers (10 to 336 fibers/kg) and fragments (from 2 to 82 fragments/kg). The presence of fibers was also observed at the plant level (on average 77.9 ± 19.0%, *n* = 27), which suggests that these foreign particles reach the level of flower nectar, where they are transferred to the hive by bees and from there end up in bee products that humans consume. When discussing synthetic organic fibers, the following are included: polyester, polyethylene, polypropylene, polyamide and polytetrafluoroethylene. Atmospheric particles can be derived from a variety of sources: sewage, clothing abrasion and the fragmentation of macroplastics in environmental conditions (oxygen, sunlight action, temperature, humidity). This raises the alarm that the environment is increasingly affected by microplastic particles, particles that end up on people’s dinner plates [148,149].

In addition to honey samples, polyethylene, polypropylene and polyacrylamide polymers were also found in beer, milk and soft drinks collected in Ecuador [149]. In 2017, researchers analyzed five honey samples from Switzerland following a standardized protocol. In the study, they used a microscope to separate fibers and fragments based on their color or transparency. However, to more precisely identify the presence of microplastics, they also used Raman spectroscopy and the FTIR method. The researchers performed procedural blanks and minimized the risk of sample cross-contamination. Most of the fibers detected were identified as cellulose, which is a natural fiber, or PET. The most likely origin is believed to be from textiles [150].

Much research has been done in recent years into the effects that microplastic contamination has on terrestrial and aquatic systems, but the impact of microplastics on bees in addition to their transfer into hive products is less clear. Unfortunately, in recent years, large losses have been recorded for bee colonies; this fact is worrying considering the ecological importance they have. There are many factors considered incriminating for colony damage (presence of pesticides, parasites, microbial infections, toxic metals) [151,152], although the potential link between the exposure of bees to microplastics and the damage that can be caused has not yet been sufficiently investigated; this is an assumption that is brought to the attention of future research [153,154].

### 5.4. Aquatic Species

Contamination of marine animals with plastic particles can occur through direct ingestion or trophic transfer. For example, when people eat small fish, they eat them whole, including the intestines where the microplastics are found; there is contamination by direct ingestion. On the other hand, trophic transfer, also known as bioaccumulation, exposes humans to chemicals released by microplastics indirectly. More precisely, these chemicals are compounds that enter into the composition of microplastics or persistent pollutants adsorbed by them (pesticides, toxic metals) [155,156,157,158,159]. These chemicals accumulate in the body from multiple sources. For example, with a crustacean contaminated by direct ingestion of microplastics, the latter may be ingested by a predator that becomes indirectly contaminated. If this predator ingests several contaminated shellfish, the chemicals from the various shellfish will accumulate in its digestive tract. This is called the phenomenon of bioaccumulation [160]. Therefore, humans may face higher exposure when consuming a marine species whose digestive tract has accumulated a high concentration of these chemical compounds.

In studies analyzing the contamination of fish with microplastics in laboratories, it should not be overlooked that these fish generally ingest high concentrations. In addition, most experiments are carried out with spherical microplastics of a predefined size. However, in the environment, microplastics come in a variety of shapes and sizes [161].

### 5.5. Impact of Plastic Waste on Marine Organisms

The path of plastic particles to an aquatic or terrestrial organism will determine the possible toxic effects. After ingestion, MPs can either block the digestive system [162] or simply pass through it (the main route observed in the laboratory, for example) [163] or, in the case of the smallest particles, pass through digestive membranes and migrate to the circulatory system or even to other organs [164,165]. MP that is not released in feces could be transferred along the food chain as experimentally demonstrated [166,167].

Ingestion of MPs in the laboratory has been observed many times with the help of histological sections or with the help of fluorescent spheres on a wide range of taxa: zooplankton [168,169], bivalves [170,171], crustaceans [172,173]. Despite cessation of exposure, MPs can persist for several days in the body without being excreted, increasing the risk of gastrointestinal obstruction and inflammation [162]. For example, in the crab *Carcinus maenas* [173], the presence of MPs was observed 14 days after cessation of exposure. Particle shape will also modulate retention time [174,175]. The smooth and perfectly spherical shape of commercially available MPs facilitates rapid transit in the digestive system of organisms [176]. For example, after 24 h of depuration, 99% of daphnia exposed to microspheres showed complete MP excretion, compared to only 1% of daphnia exposed to micro-fragments [174].

Translocation corresponds to the passage of microparticles or nanoparticles through the epithelia of an organism to the circulatory system and to different organs and tissues by crossing different membranes [177]. Experimentally, the translocation of plastic microspheres has been observed in the circulatory and lysosomal system of bivalves [170,178], in the liver of zebrafish *Danio rerio* [179] or in the hepato-pancreas of crabs [177,180,181]. For example, visualization of MPs in the hemolymph of bivalves is performed by sampling with a syringe through the mantle; it is recognized that MPs can adhere and accumulate on external organs (e.g., mantle, leg) [182].

Theoretically, the translocation efficiency mainly depends on the particle size. In the absence of previous damage to the digestive tract, the risk of MPs passing through the epithelium of the digestive tract of marine organisms is low, for example, in fish [183]. No physiological effects were found on the rainbow trout gut (e.g., permeability, ion and amino acid transport) after 4 weeks of exposure (PS; 100–400 μm) [184]. The use of MPs in medical studies demonstrated low translocation efficiency (translocation efficiency < 0.05%) of particles <5 μm, while this translocation efficiency was 0.2–10% for NPs [185,186]. As the size decreases, the surface-to-volume ratio and reactivity of the particles increases, facilitating membrane interactions and barrier crossing [187,188,189]. Depending on the surface properties, the ability of NPs to cross barriers changes. Indeed, membrane interactions are facilitated for positively charged particles due to attraction processes with negatively charged membrane residues [190]. For example, polystyrene nanospheres positively charged at amino groups (-NH_2_) (50 and 100 nm) show a higher rate of internalization inside mammalian cells compared to particles negatively charged at carboxyl groups (-COOH) [191]. Three types of passage mechanisms may be involved: endocytosis, phagocytosis and passive membrane exchange [192,193].

In the marine domain, NP translocation has been observed experimentally in several fish species [194,195,196], in clams [164] and in daphnia [197,198]. The potential passage of MPs (<10 μm) and NPs could be promoted or reduced depending on the chemical nature of the eco- or bio-corona via membrane recognition mechanisms [177,187,199,200]. In the case of translocation, the retention time would increase, and the release of the particles would prove difficult. Thus, in accordance with their experimental data, Al-Sid-Cheick et al. (2018) estimate the annual bioaccumulation of NPs (24 nm) by *Pecten maximus* at 123 μg NP g^−1^, 12 μg NP g^−1^ and 1.8 mg NP g^−1^ for a constant concentration of 1 μg L^−1^, 100 μg L^−1^ and 15 μg L^−1^, respectively [164].

Based on laboratory exposures, ingestion of MPs is rarely lethal to exposed organisms. However, the accumulation or simple transit of these particles in the digestive tract and external organs (e.g., gills) can cause disturbances at different scales: molecular, cellular, individual and population [187,201]. It should be noted that in some cases the effects observed after exposure to plastic particles could be associated with the release of chemical compounds (e.g., additives), but this remains to be further studied [202].

Disturbance of the oxidative system of the organism infested with microplastics, i.e., the defense system against external stress, is one of the major processes observed in the specialized literature [171,203]. For example, Jeong et al. (2017) observed the stimulation of molecular pathways (e.g., MAPK—the activation of mitogen-activated protein kinase) associated with the defense of the copepod *Paracyclopina nana* after 24 h of exposure to PS nanospheres (50 nm) [203]. This oxidative stress can be associated with dysregulation of the immune system [178,199,204], stimulation of molecular pathways related to apoptosis [205,206], membrane damage (e.g., lipid peroxidation, loss of integrity) [179,207,208], destabilization of the lysosomal system [14] or genotoxicity (e.g., loss of DNA integrity, DNA breaks) [209,210]. Omic methods (e.g., transcriptomics, proteomics, metabolomics) allow unbiased characterization of a set of molecular pathways involved during exposure and are therefore to be preferred in future studies [202,204,211,212] to understand the response of the organism exposed to plastics and also to explore resilience mechanisms.

Various tissue reactions can occur following MP ingestion. Thus, necrosis, increased mucus production, inflammation and damage to the intestine were observed [213,214]. For example, the brain of the freshwater fish *Carassius carassius* showed various lesions (e.g., discoloration, swelling) following exposure to PS nanospheres (30 nm) [211], which could explain the observed behavioral changes.

Ingestion and simple transit of MPs can influence the energy metabolism of individuals. One of the first effects of MP ingestion is that of satiety, changing the body’s eating behavior. Thus, in most cases, a reduction in food consumption is observed [215,216,217,218]. However, the opposite phenomenon was already observed in oysters (a 3% increase in filtration activity) after two months of exposure to PS microspheres (2 and 6 μm), probably as part of an energy compensation mechanism [202]. The effect on food consumption may depend on particle size depending on the species. A 70% decrease in algal consumption was found in *Crassostrea gigas* larvae exposed to 1 μm particles but no effect was found with 10 μm particles [219]. In addition, there was also no difference between the effects of 2 μm microspheres and 100 nm nanospheres on food consumption in *Daphnia magna* [216]. This disruption of food consumption, and therefore of reserve accumulation, can lead to an effect on growth, metabolic activity or reproductive effort to maintain organisms [203,220,221,222,223]. For example, analyzing the energy budget of oysters, Sussarellu et al. (2016) highlighted an increase in maintenance energy at the expense of reproductive effort (e.g., the amount of gametes produced) [202]. This modulation of energy balance was also observed under NPs with a size- and dose-dependent effect [218]. For example, PS nanospheres (50 nm; 20 μg mL^−1^) induced a 59–70% decrease in fecundity (number of fry produced) of the rotifer *Brachionus koreanus* and the copepod *Paracyclopina nana*, while no effect for microspheres (6 μm) was observed [224].

The presence of MPs can induce changes in the behavior of organisms directly or as a result of cellular/technological/energetic perturbations [187]. In the laboratory, MP ingestion has been shown to reduce the jumping ability of sea fleas [225], alter fish locomotion/feeding (e.g., distance traveled, feeding time) [211,226,227] or induce daphnia immobilization [228]. These behavioral changes can impact prey–predator interactions. Additionally, depending on particle shape (e.g., sphere, fiber), zooplankton species may alter their feeding preferences to limit the risk of ingesting plastic particles with a shape similar to that of natural prey [169,229].

Community stability and ecosystem functioning are related to the ability of populations to maintain and acclimatize to anthropogenic disturbances. Most ecotoxicological studies focus on effects over a single generation, but assessing ecological risk to marine ecosystems requires exploring the effects of a contaminant over multiple generations. To date, four studies have examined MP effects across at least two distinct generations [230,231,232]. Regarding MPs, Zhang et al. (2019) observed no consequence of exposure to generations 1 and 2 of *Tigriopus japonicus* on generation 3 [233]. However, in the same species, Lee et al. (2013) observed higher toxicity of nanospheres (50 and 500 nm, respectively) in the first generation, as well as increased sensitivity to NPs (50 nm) in generation 2 [230].

Most of the studies in the specialized literature evaluated the toxicity of MPs on organisms in the adult stage [234]. However, many marine organisms (e.g., bivalves, sea urchins) have a mode of reproduction by external fertilization with a pelagic larval life. Thus, the young stages (gametes, embryos, larvae) are in direct contact with plastic debris from the environment.

Since the toxicity of MPs is mainly related to their ingestion and disruption of energy balance, it seems unlikely that these particles have an impact on gametes and embryos of marine organisms. On the other hand, due to their size and possible translocation capacity, NPs are more likely to interact with these young life stages. There are no available data on the effect of NPs on spermatozoa and oocytes, while in assessing the impact of different contaminants (e.g., metal nanoparticles, toxic algae), gametes have been shown to be very sensitive stages [235,236,237,238]. Regarding the effects on the embryonic development of marine species, a dose-dependent toxicity of NPs (PS 50 nm) was observed in the mussel *Mytilus galloprovincialis* (EC50—half maximal effective concentration = 0.14 μg mL^−1^) [239] and the sea urchin *Paracentrotus lividus* (EC50—half maximal effective concentration = 2.61 μg mL^−1^) [205], associated with the modulation of the molecular pathways of apoptosis for the sea urchin and shell mineralization for mussels. Ingestion of MPs by larval stages of different species has been demonstrated in natural [240] or controlled environments [241,242]. However, most studies have seen a limited effect, even at very high doses. For example, in two different works [243,244], chronic exposure (8 days (a) and 15 days (b) of *Crassostrea gigas* larvae to MPs (a) 1 and 10 μm, 100 MPs mL^−1^ and (b) 2 and 6 μm, 2000 MPs mL^−1^ had no effect on larval growth or food consumption. The same result was observed in the mussel *Mytilus edulis* with 2 μm particles after 15 days of exposure (42–282 μgL^−1^) [243]. In contrast, in the same study, larvae exposed to NPs (100 nm) showed numerous malformations at the end of exposure [244]. Behavioral changes, neurotoxic and cellular effects (e.g., decreased energy production, disruption of cardiac activity and antioxidant system) were also observed in larvae of *Artemia franciscana* [245] and *Danio rerio* [227,246,247] after NP exposures. Thus, while larvae appear to be little affected by MPs, nanoparticles could have superior effects on these stages, mainly due to their reactivity and size favoring interaction with biological membranes and the development of lesions.

### 5.6. Impact of Plastic Waste on Human Health

Today, the main talk is about the physical effects of plastic debris on marine animals. Indeed, these animals can be endangered by old fishing nets or plastic bags. As a result, they no longer know how to move to feed or avoid predators and sometimes even come to the surface to breathe. Others may be so exhausted that they sink or cut, causing an infectious process later. Numerous studies report that large numbers of marine animals ingest macroplastics. A recent study reveals that 100% of sea turtles have plastic waste in their digestive systems. The same is true for 59% of whales, 36% of sea lions and 40% of birds [248]. This can cause a satiety effect when nutrient intake is not sufficient. On the other hand, pieces of plastic can obstruct the intestinal tract. All this can lead to the death of the animal due to malnutrition and deterioration of health.

The physical effects caused by microplastics are less studied. However, there is evidence that small particles can attach to the internal or external surface of marine organisms. This can lead to physical damage that causes stress or inflammation, or even go so far as to block the absorptive surface of the intestinal lining. Consequently, these organisms experience a decrease in energy intake. However, no studies report that this information is transferable to humans.

Unlike macro- and mesoplastics, which mainly have physical effects on wildlife, microplastics have the potential to have chemical effects. They would be considered vectors of persistent chemical pollutants [249].

Plastic is mostly made up of synthetic polymers. They are made up of a chain of monomers. There is a wide variety of synthetic polymers, and the most widely used is polyethylene. It constitutes more than 40% of the plastic materials produced. Low-density polyethylene (LDPE) is used in food films, plastic bags, agricultural films and many other materials and packaging. High-density polyethylene (HDPE) is harder and more opaque, and more resistant to tension and higher temperatures (120 °C) than LDPE. It is used for the manufacture of toys, crates and boxes, bottles for food products, detergents, cosmetics, etc. The second most used polymer worldwide is polypropylene (PP). It is less chemically resistant but has better thermal and mechanical properties. Plastic bottles are mainly made from the polymer polyethylene terephthalate (PET). In turn, synthetic clothing fibers are mainly made of polyester but also of acrylic fibers [250].

All these polymers are included in the composition of microplastics. During the degradation of these microplastics, residual monomers can be released. They are a known hazard to human health. For example, polyurethanes and polyvinyl chloride have carcinogenic and mutagenic effects in humans. First, I3A (indole-3-aldehyde) is known to be a metabolite of dietary L-tryptophan that is synthesized by gastrointestinal bacteria, especially species of the genus *Lactobacillus* [251]. I3A acts as an agonist of the aryl hydrocarbon receptor (AhR) in intestinal immune cells and in turn stimulates the production of interleukin-22 (IL22), which facilitates mucosal reactivity [252]. Furthermore, I3A is an indole derivative that triggers glucagon-like peptide-1 (GLP-1) secretion in intestinal L cells and acts as a ligand for the AhR. Indole can also be metabolized in the liver to indoxyl sulfate, a compound that is toxic at high concentrations and associated with vascular disease and renal dysfunction. Beyond this, I3A causes cell differentiation and apoptosis. It up-regulation results in the attenuation of liver fat and promotes remission of non-alcoholic fatty liver disease (NAFLD) [253], while its down-regulation promotes the formation and worsening of NAFLD. A significant decrease in I3A levels in the liver was observed after one week of exposure to microplastics, suggesting that the ability of intestinal tryptophan to convert and transport I3A to the liver is impaired. This is potentially associated with the production of indole sulfate, a toxicant associated with oxidative stress in the liver [254].

**Figure 6:**
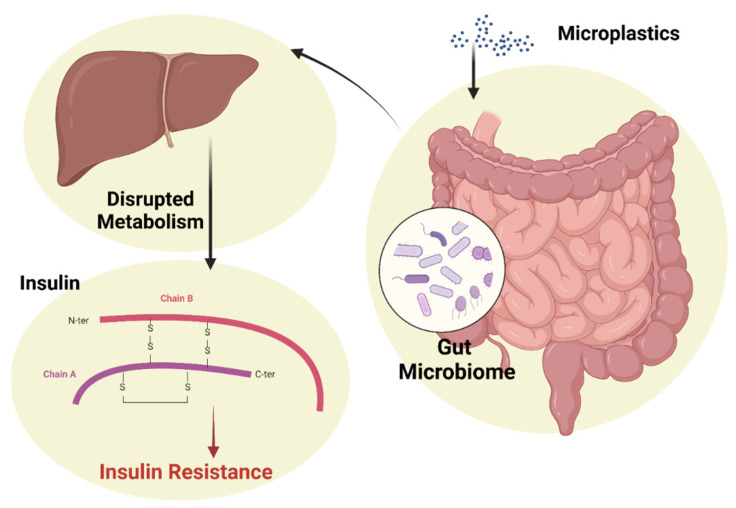
Insulin resistance and exposure to microplastics. Created with BioRender.com.

The effects of ingesting microplastics have been identified and classified by researchers into three stages: the first is related to the blockage and damage of the digestive system, the second refers to the release of toxic chemicals into the body, and the third stage is represented by the assimilation of these substances by organs and tissues. Due to the increased human exposure to microplastics, they can be absorbed into the body through various pathways and accumulate in organs such as the liver, kidneys and intestine. Scientific studies have found that exposure to microplastics causes intestinal inflammation and liver metabolic disorders, but it is not yet known whether the damage and inflammation can cause the subsequent development of serious diseases. What the mouse study found is that daily exposure to microplastics has effects on the gut–liver axis, effects that ultimately lead to insulin resistance and even diabetes (Figure 6). These results indicate the urgent need regarding the prognosis of insulin resistance after exposure to microplastics [255].

Overwhelming of antioxidant responses may result in oxidative stress. Microplastics may be at the origin of this oxidative stress, caused by their high surface area, release of oxidizing species adsorbed to their surface (e.g., metals) or due to reactive oxygen species released during the inflammatory response [256,257]. For instance, oxidative stress after exposure to microplastics has been reported in mice [258]. In polypropylene (PP) prothesis, after insertion acute inflammatory response culminates with the release of oxidants (e.g., hydrogen peroxide, hypochlorous acid) inducing degradation, hydrolysis, cracking and additive leaching of the polymer producing a positive feedback loop of free radical production and revealing potential mechanisms of plastic removal from the organism [259]. Cytotoxicity is a result of particle toxicity, oxidative stress and inflammation. Cellular internalization of microplastics has been described for PS (polystyrene) in cell cultures, including macrophages, erythrocytes [260] and rat alveolar epithelial cells [261]. Inside the cell, microplastics are not membrane bound, potentially interacting with intercellular structures [260]. In vitro testing has been able to show cytotoxicity caused by plastic particles collected from the environment [262]. On the other hand, exposure to 0.05–10 mg L^−1^ of PS and polyethylene in cerebral and epithelial human cells was not able to induce cytolysis but increased reactive oxygen species (ROS) to high concentrations, contributing to cytotoxicity [263]. Furthermore, exposure of macrophage and lung epithelial cell cultures to PS (60 µm) caused ROS and endoplasmic reticulum stress (caused by the aggregation of misfolded proteins) leading to autophagic cell death [264]. Thus, cytotoxicity and oxidative stress may be important mechanisms of microplastic toxicity.

Exposure to contaminants may lead to neurotoxicity, which is related to neurodegenerative diseases. Neurotoxicity has been reported in vivo after exposure to particulate matter, possibly due to oxidative stress and the activation of the microglia in the brain (immune cells) due to direct contact with translocated particles or through the action of circulating pro-inflammatory cytokines (from other inflammation sites), resulting in damage to neurons [265]. Indeed, exposure to traffic pollution, including particulate matter, has been associated with mild cognitive impairment in the elderly, increasing the risk of Alzheimer’s disease development [266] and higher incidence of dementia [267]. Through the same mechanisms, and depending on individual susceptibility, microplastics could contribute to the increasing incidence of neurodegenerative diseases. Indeed, in vivo toxicity testing has shown that microplastics can impact neuronal function and behavior. In the brain of European seabass (*Dicentrarchus labrax*), microplastics are reported to cause inhibition of acetylcholinesterase (AChE), oxidative stress with increase in lipid peroxidation levels and an increase in the anaerobic pathway of energy production [268]. In the same species, exposure to microplastics has been shown to impact swimming performance, a behavioral indicator [269]. Exposure to PS has also been reported to cause adverse effects on neurotransmission in mice, such as increased activity of AChE and changes in serum neurotransmitters [258]. In in vitro studies using neural cell types, 40–70 nm PS nanospheres were also able to induce toxicity and changes in metabolic activity, dependent on cell-type and concentration, with increased toxicity after long in-shelf storage periods of PS due to increased aggregation and presence of bioactive compounds [270]. Due to the evidence of neurotoxicity when testing microplastics in organism or cells and resulting from human exposure to particulate matter, which microplastics are a part of, there is a need to understand how microplastics could be involved in neurotoxicity in humans, contributing to an increased risk of neurodegenerative disease development [271,272].

Although microplastics may not have major immediate effects, they likely potentiate or exacerbate many medical conditions, especially respiratory problems. An inflammatory gene, TRPV4, that was up-regulated by microplastics is activated in critically ill COVID-19 patients, causing lung edema, a condition of respiratory distress found in workers exposed to microplastics in the plastic manufacturing industry [273,274]. The cell-to-cell loss of adhesion we report here is likely the first step of the dysfunction seen by others in cell and tissue barriers in vivo on exposure to microplastics, providing further evidence that long-term exposure to the inhalation of PS-MPs presents a serious hazard to lung health, while short-term health has not been found to be endangered by microplastics except at high concentrations in industrial settings [275].

During the production of plastic, additives are usually added. They would constitute on average 4% of plastic. These additives can be antioxidants, pigments and stabilizers, and perform various functions such as increasing the flexibility, durability and transparency of the plastic. Additives include phthalates, bisphenol A and PAHs (polycyclic aromatic hydrocarbons). Bisphenol A is also the monomer used in the manufacture of polycarbonate. During the fragmentation of microplastics, additives can migrate more easily from the center of the polymer to the surface [105]. Therefore, the size of the microplastic may play an important role in the release of chemicals [109].

This release of additives into the marine environment worries scientists. Indeed, these chemicals can cause harmful effects to the health of marine animals and humans. Certain additives such as phthalates and bisphenol A are endocrine disruptors. These endocrine disruptors work through different modes of action, such as binding to natural hormone receptors. For example, bisphenol A is an estrogen mimetic, meaning it binds to alpha and beta estrogen receptors. This can lead to reproductive disorders. EFSA (European Food Safety Authority) has established the tolerable daily dose of bisphenol A: 0.004 mg/kg body weight/day. Therefore, people should be exposed to a lower dose than this to avoid harmful problems to their health. Endocrine disruptors can also act in other ways, either by mimicking the action of a natural hormone or by antagonizing the production or regulation of hormones or receptors.

**Figure 7:**
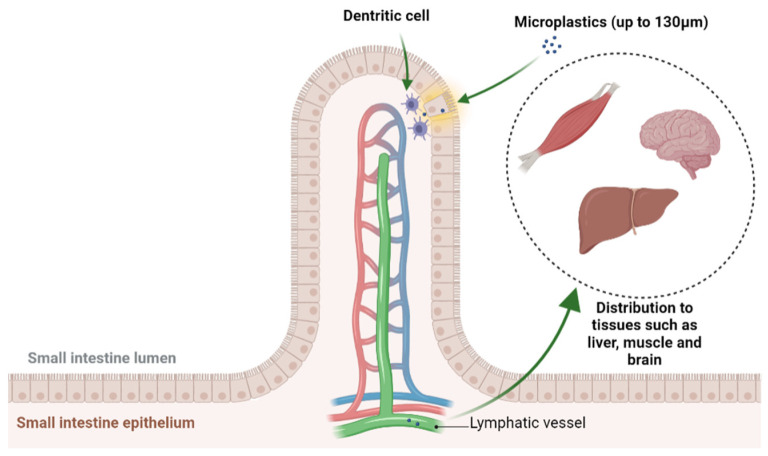
Crossing of microplastics in the digestive tract. Created with BioRender.com.

However, these particles could be absorbed in the digestive tract by other transport mechanisms. In the study by Wright and Kelly (2017), two hypotheses are presented: uptake by endocytosis and absorption. Endocytosis is thought to be carried out by M cells of Peyer’s patches, mainly in the ileum. Microplastics between 0.1 and 10 µm would therefore be transported from the intestinal lumen to the lymphoid tissues of the digestive tract [110]. Persorption would consist of paracellular transport, i.e., absorption of particles smaller than 130 µm between two cells. This would occur mainly in unstratified epithelia and where there is junctional weakness between cells (Figure 7). Dendritic cells would then phagocytose the particles and transport them to the lymphatic vessels and portal vein. Therefore, there would be a possible distribution to tissues such as liver, muscle and brain [105].

The major problem lies in assessing the risks to human health. No studies have yet been conducted on the possible accumulation and toxicological effects of microplastics after the ingestion of these foods. No threshold has been established to ensure that there is no risk to humans [94].

Although conscious consumers are encouraging the reduction of single-use plastics, some manufacturers are creating new plastic packaging to replace traditional uses of paper, such as plastic tea bags. Particle levels of nylon and polyethylene terephthalate released from tea bag packaging are also a concern; an initial assessment of acute toxicity in invertebrates shows that exposure to particles released only from tea bags caused dose-dependent developmental and behavioral effects [276].

## 6. Solutions to Reduce Microplastic Pollution

With today’s technological means, it is impossible to eliminate the microplastics present in the environment. Therefore, it is necessary to act rather at other levels, i.e., at the level of macro and mesoblasts.

The field of bioplastics has emerged to solve some of the many problems with conventional plastics. Bioplastics include biobased plastics and biodegradable plastics [277]. In the case of bioplastics, non-renewable sources of plastic monomers have been replaced by renewable sources.

For example, in bio-PE, the ethylene monomer in the plastic is produced from sugar cane starch instead of petrochemicals. However, although the shift to plant sources has a positive impact by reducing the demand for petrochemicals, there are other problems such as deforestation, increased use of pesticides and the need for extensive chemical processing. Biomass-based plastics do not differ from conventional ones in terms of their properties and contain chemical additives similar to those in conventional plastics. Unlike conventional plastics, biodegradable plastics can break down into molecules of water and carbon dioxide, and compost under certain circumstances in the environment through the action of microorganisms. There is no time limit for plastics to become biodegradable; the process can take months. Unless the right circumstances are met, biodegradable plastics will not degrade and end up contaminating like regular plastics. Biodegradable plastics can be made from non-renewable fossil sources or renewable resources such as wood, crops and food waste, and are typically used in short-lived applications such as food packaging, disposable tableware and some agricultural applications.

In general, the field of bioplastics reflects the need for the plastics industry to move towards more environmentally sustainable solutions. The need for an innovative solution to reduce this pollution is inevitable. Increasing the recycling of plastic waste is not a comprehensive solution in itself. In addition, reducing the use of fossil-based plastic is an important aspect of sustainability. As an alternative to fossil-based plastics on the market, biobased plastics are increasingly popular. According to the studies carried out, products with similar performance characteristics can be obtained using biological raw materials instead of fossil sources. In particular, the production of bioplastic from microalgae (Chlorella and Spirulina species were the most commonly used in the production of both biopolymers and plastic blends) is a new opportunity to be further explored and improved [277].

### Legislative Regulations for Limiting Pollution with Microplastics

In recent years, more and more regulations have been developed that target the production of plastic but also its transport, commercialization, collection and recycling, trying as much as possible to eliminate/reduce the pollution of the environment of aquatic organisms with plastic masses. The long-term assurance of human health is desired, as well as a legacy as little as possible contaminated with plastic products for future generations.

The increase in plastic pollution has generated discussions among regulators, scientists and the general public about how this problem affects ecosystems and human health. The dangers of microplastic contamination are just beginning, and action is not yet being taken seriously by all. The proposed regulations require attention but must be as applicable as possible so that the results of implementation can be observed in the shortest possible time [278].

In the United States, the Microbead-Free Waters Act, which primarily targets microplastic pollution and, by extension, plastic bag pollution, prohibits both the manufacture, packaging and distribution of rinse-off cosmetic products containing plastic particles. The regulation applies to both cosmetics and over-the-counter (OTC) drugs such as toothpaste. The use of products that are biodegradable alternatives to plastic is promoted [279]. In the United Kingdom, the Commonwealth Clean Oceans Alliance was established with the goal of eliminating single-use plastic. The Commonwealth Clean Oceans Alliance is an agreement between the United Kingdom and several countries (Ghana, Sri Lanka, New Zealand and Vanuatu), which addresses the issue of marine plastic. Signatory countries have committed to ban microplastics, cosmetics and rinse-off products and reduce the use of plastic bags. This Alliance aims to collaborate with big brands in order to reach the proposed goals in the shortest possible time but also through the most effective means [280,281].

In order to encourage the population to take part in a project to reduce plastic pollution, the Plastic Bank proposes for the inhabitants of Haiti, Brazil and the Philippines to bring plastic waste to areas set up for their collection; the Bank then rewards residents with digital tokens that can be exchanged for goods such as water, food or phone minutes [282]. In China, the Law on the Prevention and Control of Environmental Pollution by Solid Wastes (LPCEPSW) regulates landfills and prohibits the dumping of plastic masses into rivers, reservoirs and lakes [283].

In France, the Circular Economy Law has banned single-use plastics; microplastics are allowed in human and veterinary medicine [279]. In Italy, the Plastic Packaging Law requires the introduction of taxes for plastic materials. The law has been postponed several times [284].

The Association of Southeast Asian Nations (ASEAN), consisting of Cambodia, Indonesia, Brunei, Laos, Malaysia, the Philippines, Myanmar, Singapore, Thailand and Vietnam adopted the ASEAN Regional Action Plan to combat marine debris in the member states of this Association. The plan aims to reduce the release/leakage of plastic into the system, ensuring an increase in the degree of water purification [282].

In 2017, the European Commission asked ECHA (European Chemicals Agency) to assess the scientific evidence for taking EU-wide regulatory action on microplastics that are intentionally added to products (i.e., substances and mixtures). ECHA has proposed a broad restriction on microplastics in products placed on the market to reduce microplastic pollution and to avoid environmental contamination as much as possible [282].

In 2018, during the G7 (Group of 7) meeting, countries such as Germany, Italy, Canada, Great Britain, France, Germany and the European Union (EU) signed the “Ocean Plastic Charter” where several policies were proposed (among which, by 2030, 100% recyclable or recoverable plastic materials should be used, the reduction as much as possible of the unnecessary use of single-use plastic materials), policies aimed at preventing the contamination of the environment with plastic masses, promoting the use of alternatives to plastic and encouraging and increasing recycling [285].

## 7. Conclusions

Microplastics are present in all ecosystems (atmosphere, soil, seas and oceans) and in many organisms (fish, birds, domestic animals and humans); therefore, corrective measures are needed at the global level to significantly reduce the use of plastic. Because of their high resistance to degradation, microplastics persist for a long time in the environment. To date, no solution has been found to extract/remove them from the environment, and the global overconsumption of plastic worsens the accumulation of these particles in these natural environments. The chemical substances that enter their composition, as well as the pollutants adsorbed and then released by microplastics, generate harmful eco-toxicological effects for the health of animals and people. However, there is a lack of experimental data to fully assess toxicity in humans, and no safety threshold for the human body has yet been established. Before we can certify that microplastics pose a danger to biota and humans, it is necessary to improve the quality and international standardization of the methods used to assess their exposure, risks and effects. In the meantime, it is strongly recommended to reduce the accumulation of these microplastics in the environment. Efforts are being made to find viable solutions to reduce the accumulation of macroplastics in the oceans but also to reduce plastic consumption globally.

## Figures and Tables

**Table 1:** Presence of microplastics in different food products.

Type of Food	Abundance Average Particles	Range Size Particles	Type of Polymer	Method of Detection	Reference
Salts from Taiwan market	9.77 MP/kg	1–1500 μm	Polyethylene terephthalate (PET), Polypropylene (PP), Polyethylene (PE)	FTIR spectroscopy	[116]
Sea salts from Bangladesh	2676 MP/kg	0.1–5 mm	Polystyrene (PS), Ethylene-vinyl acetate (EVA), High-density polyethylene (HDPE), Nylon (polyamide 6), Polyethylene terephthalate (PET)	FTIR spectroscopy	[117]
Salt from India (Gujarat) (Tamil Nadu) (Parangipettai, Marakkanam)	46–115 particles/200 g 23–101 particles/200 g 5–21 particles/10 g	100–1000 µm 100–1000 µm 100–1000 µm	PE, PVC (Polyvinyl chloride), PS PE, PVC, PS LDPE (Low-density polyethylene), PP, PET, Nylon	FTIR spectroscopy FTIR spectroscopy FTIR spectroscopy	[118] [118] [119]
Table salt from Spain (21 different samples)	50–280 particles/kg	10–3500 µm	PET, PP, PE	FTIR spectroscopy	[120]
Sugar from Germany	249 ± 130 particles/kg	<0.8 µm	PET, PE, PP	Dissection microscope	[121]
Glass bottled water Reusable plastic bottled water Single use plastic bottled water from Germany	35,436 MP/L 23,594 ± 25,518 MP/L 2649 ± 2857 MP/L	90% < 5 µm	PET, PE, PP	Micro-Raman spectroscopy using an XploRa Plus system, operated by LabSpec 6 software (Horiba Scientific)	[122]
Raw water from Germany	0–7 MP/m^3^	50–150 μm	PE, PA(Polyamide), PS, PVC	FTIR imaging	[123]
Drinking water from Saudi Arabia	1.9 ± 4.7 particles/L	25–500 µm	PE, PS, PET	FTIR microspectroscopy	[124]
Plastic food containers from China	1–41 MP per container	≤500 μm; 501–100 μm; ≥1001 μm;	PS, PP, PE, PET	SEM (scanning electron microscope), µ-FTIR	[125]
Plastic food tray with sealing film from France	4.0–18.7 MP/kg	<1 mm	XPS (Extruded polystyrene)	FTIR	[126]
Single use PET bottles from China	2649 ± 2857 MP/L	5–10 μm 1.7% MP; 1.5–5 μm 44.7% MP; ≤1.5 μm 53.6% MP	PET, PP, PE, PET + olefin	Micro-Raman Spectroscopy	[127]
Tea bags from Canada	11.6 billion MP per cup of tea beverage	10 nm–150 µm including nanoparticles	PET, Nylon	SEM, FTIR	[128]
Infant feeding bottles from China	16.2 million MP/L	1–20 μm	PP	Raman spectroscopy, atomic force microscopy	[129]
Bivalve mollusks Crustaceans from South Korea	0.15 ± 0.20 particles/g 0.97 ± 0.74 particles per individual	43–4720 µm 65% < 300 µm	PE, PP, PS, PES (Polyethersulfon)	µ-FTIR (micro-Fourier transform infrared microscope)	[130]
Bivalve mollusks and crustaceans from China	0.5–3.3 particles per individual	7–5000 µm	CPE (Chlorinated polyethylene), PET, PVDF (Polyvinylidene fluoride), PVDC (Polyvinylidene chloride), PE, PVE (Polyvinyl ethers), Nylon, PE, PEI (Polyethylenimine), PAN (Polyacrylonitrile), PVC, CPE (Chlorinated polyethylene), Rayon	μ-FTIR	[131]
Fish (*Siganus rivulatus, Diplodus sargus, Sardinella aurita, Sphyraena viridensis, Atherina boyer*) from Egypt	28–7527 particles/fish	≤25–2000 µm	PVA (Polyvinyl alcohol), LDPE, HDPE, PET, PP, Nylon	FTIR spectrometry, Raman spectroscopy, HT-GPC (High-Temperature Gel Permeation Chromatography)	[132]
23 milk samples (22 for adult and 1 for child) from Mexico	6.5 ± 2.3 particles/L	0.1–5 mm	PES, PSU (Polysulfone)	SEM, Raman spectroscopy	[133]
Honey from Switzerland	32–108 Fibers/kg	30 and 1 μm	PET	Raman and Fourier transform infrared spectroscopy	[134]
Canned sardines from Australia and Malaysia	1–3 fragments per individual	149 and 8 μm	PP, PET	Micro-Raman spectroscopy, Energy-Dispersive X-ray spectroscopy (EDX)	[135]
Fruits (pear, apple) Vegetables (lettuce, broccoli, carrot) Food and beverage packaging from Italy	52,600–307,750 MP/kg 72,175–130,500 MP/kg	1.81–2.29 μm 1.51–2.52 μm	Not specified Not specified	Scanning electron microscopy (Cambridge Instruments Mod. Stereoscan 360) combined with an X-ray Energy Dispersion Detector (SEM-EDX)	[136]

## Data Availability

Not applicable.
